# StarPepWeb: an integrative, graph-based resource for bioactive peptides

**DOI:** 10.1093/bioadv/vbaf261

**Published:** 2025-10-16

**Authors:** Christian López, Roberto Cárdenas, Longendri Aguilera-Mendoza, Guillermin Agüero-Chapin, Félix Martínez-Rios, César R García-Jacas, Noel Pérez-Pérez, Yovani Marrero-Ponce

**Affiliations:** Colegio de Ciencias e Ingenierías “El Politécnico”, Universidad San Francisco de Quito USFQ, Quito 170157, Ecuador; Colegio de Ciencias e Ingenierías “El Politécnico”, Universidad San Francisco de Quito USFQ, Quito 170157, Ecuador; CIIMAR—Centro Interdisciplinar de Investigação Marinha e Ambiental, Universidade do Porto, Porto 4450-208, Portugal; CIIMAR—Centro Interdisciplinar de Investigação Marinha e Ambiental, Universidade do Porto, Porto 4450-208, Portugal; Facultad de Ingeniería, Universidad Panamericana, Ciudad México 03920, Mexico; Investigador por México, Secretaría de Ciencia, Humanidades, Tecnología e Innovación (Secihti), Ciudad de México, Mexico; Tecnológico Nacional de México, Instituto Tecnológico de Mérida, Unidad de Posgrado e Investigación, Mérida 97000, Mexico; Colegio de Ciencias e Ingenierías “El Politécnico”, Universidad San Francisco de Quito USFQ, Quito 170157, Ecuador; Facultad de Ingeniería, Universidad Panamericana, Ciudad México 03920, Mexico; Universidad San Francisco de Quito, Grupo de Medicina Molecular y Traslacional (MeM&T), Escuela de Medicina, USFQ, Quito 170157, Ecuador

## Abstract

**Motivation:**

The rapid growth of bioactive peptide sequences presents challenges for organization and analysis. Existing repositories often specialize in functions, taxonomic origins, or structural classes, but most remain isolated, use heterogeneous metadata, and lack uniform descriptors or structural models. Few integrative web services exist, offering only partial coverage or depth. As a result, reproducible and comprehensive exploration of the bioactive peptide landscape remains limited, underscoring the need for a unified, source-tracked, extensible platform.

**Results:**

We present StarPepWeb, a freely accessible web application that democratizes access to StarPepDB, one of the largest curated repositories of bioactive peptides. The platform integrates 45 120 non-redundant sequences from 40 public databases into a source-tracked graph enriched with metadata, physicochemical features, and predicted 3D structures from ESMFold. Each peptide is represented with ESM-2 embeddings and iFeature descriptors, while the interface supports metadata-aware filtering, alignment-based similarity searches with single and multiple queries, and interactive visualization. A microservice-oriented architecture ensures scalability, maintainability, and reproducible versioned downloads, including Neo4j exports. StarPepWeb thus overcomes deployment and expertise barriers of the standalone database, providing an extensible, cloud-hosted framework for integrative bioactive peptide analysis.

**Availability and implementation:**

StarPepWeb is freely available at https://starpepweb.org. Source code and documentation are hosted at https://github.com/starpep-web.

## Graphical Abstract

**Figure:**
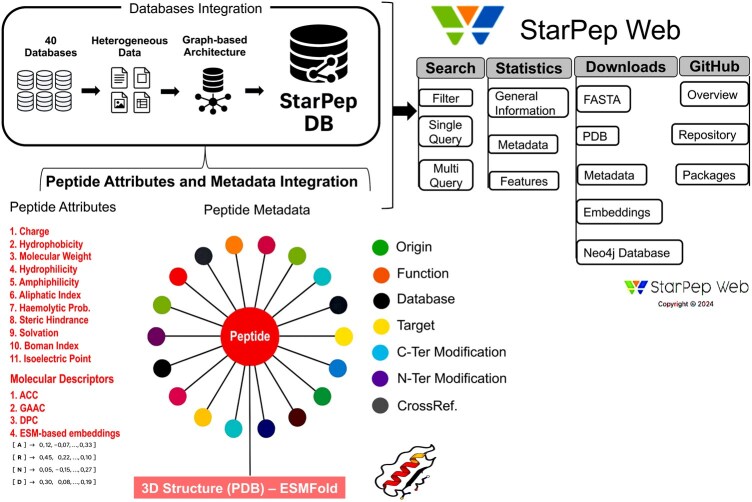

## 1 Introduction

Bioactive peptides are short amino acid sequences that exhibit diverse biological activities, including antimicrobial, antibiofilm, anticancer, tumor-homing, immunomodulatory, cell–cell communication, antioxidant functions, among others. They are derived from natural sources or can be designed synthetically. Their therapeutic applications are of increasing interest due to their high specificity, relatively low toxicity, and ability to target a wide range of biological processes (Akbarian *et al.* 2022). Currently, >100 peptide-based drugs have received FDA approval, with many more in various stages of clinical development (Al Musaimi 2024).

The vast accumulation of bioactive peptide sequences across literature and databases presents challenges in data organization and analysis (Aguilera-Mendoza *et al.* 2019). Several specialized resources focus on particular functions—such as antimicrobial [APD3, CAMP, DBAASP, DRAMP, dbAMP (Waghu *et al.* 2016, Wang *et al.* 2016, Pirtskhalava *et al.* 2021, Jhong *et al.* 2022, Shi *et al.* 2022)], antibiofilm [BaAMPs (Di Luca *et al.* 2015)], anticancer [CancerPPD (Tyagi *et al.* 2015)], and tumor-homing activities [TumorHoPe (Kapoor *et al.* 2012)]—or on taxonomic origins, including bacterial (BACTIBASE, BAGEL) (de Jong *et al.* 2006, Hammami *et al.* 2010), plant [PhytAMP, PlantPepDB (Hammami *et al.* 2009, Das *et al.* 2020)], and anuran sources [DADP (Novkovic *et al.* 2012)]. Others address structural classes such as cyclopeptides [CyBase and CyclicPepedia (Mulvenna *et al.* 2006, Liu *et al.* 2024)]. Despite this large number of databases, many still operate in isolation with heterogeneous metadata formats, which limits integrative analyses and comprehensive mining of bioactive peptides.

Relatively few web services provide an integrative and comprehensive view of the bioactive peptide landscape. Notable examples include Peptipedia (Cabas-Mora *et al.* 2024), and the Database of Food-derived Bioactive Peptides (DFBP) (Qin *et al.* 2022). While Peptipedia offers broad coverage supported by numerous activity classifiers and DFBP provides depth in a specific domain, platform-wide provision of uniform structural models and numerical descriptors for every sequence within a single, non-redundant, source-tracked resource remains limited across existing tools.

To address these gaps, we launched StarPepWeb, an effective graph-based framework for integrating heterogeneous peptide data. StarPepWeb harmonizes entries from 40 public peptide databases into a curated collection of 45 120 nr bioactive peptides, including 22 642 antimicrobial peptides (AMPs). Each peptide is represented as a node linked to detailed metadata (origin, function, sequence, source, target, types of modification and literature references) and to additional attributes such as physicochemical features and molecular descriptors (Aguilera-Mendoza *et al.* 2019). Unlike previous resources, StarPepWeb also offers precomputed ESMFold structures and ESM-2/iFeature descriptors for all peptides. These are unified in a single, nonredundant, source-tracked graph with metadata-aware filtering, single- and multi-query similarity searches, and versioned bulk downloads (including a Neo4j export).

Although StarPepWeb builds upon the standalone StarPepDB (Aguilera-Mendoza *et al.* 2019)—successfully used in hemolytic and antiviral prediction (Castillo-Mendieta *et al.* 2024, de Llano Garcia *et al.* 2024), and in the exploration of tumor homing, antibiofilm and antiparasitic chemical spaces (Ayala-Ruano *et al.* 2022, Romero *et al.* 2022, Aguero-Chapin *et al.* 2023)—the detached StarPepDB requires local deployment and technical expertise. StarPepWeb overcomes these barriers by delivering a user-friendly web application with dynamic metadata filtering, advanced similarity search and interactive data visualization. The platform is implemented with a microservice-oriented architecture to support scalability and maintenance. Therefore, this article presents StarPepWeb as a comprehensive, extensible platform for the integrative analysis of bioactive peptides.

## 2 Methods

### 2.1 Data sources and integration

StarPepWeb extends the capabilities of the standalone StarPepDB (Aguilera-Mendoza *et al.* 2019), integrating peptides from 40 public databases into a curated, non-redundant (nr) resource annotated with per-entry metadata (functional categories, taxonomic origins, targets, modifications), physicochemical properties and provenance (original source database and linked publications). For every sequence, the platform hosts precomputed models/features: ESMFold-predicted 3D models (downloadable in Protein Data Bank, PDB format), ESM-2 embeddings, and iFeature-derived descriptors (AAC, DPC, GAAC) generated offline (Chen *et al.* 2018, Lin *et al.* 2023). We selected ESMFold (MSA-free, fast inference) and ESM-2 to enable uniform, platform-wide models/embeddings at reasonable cost. These graph-structured datasets and numerical features underpin the platform’s filtering, similarity and visualization modules described in Section 2.2.

### 2.2 Implementation

**Figure 1.**
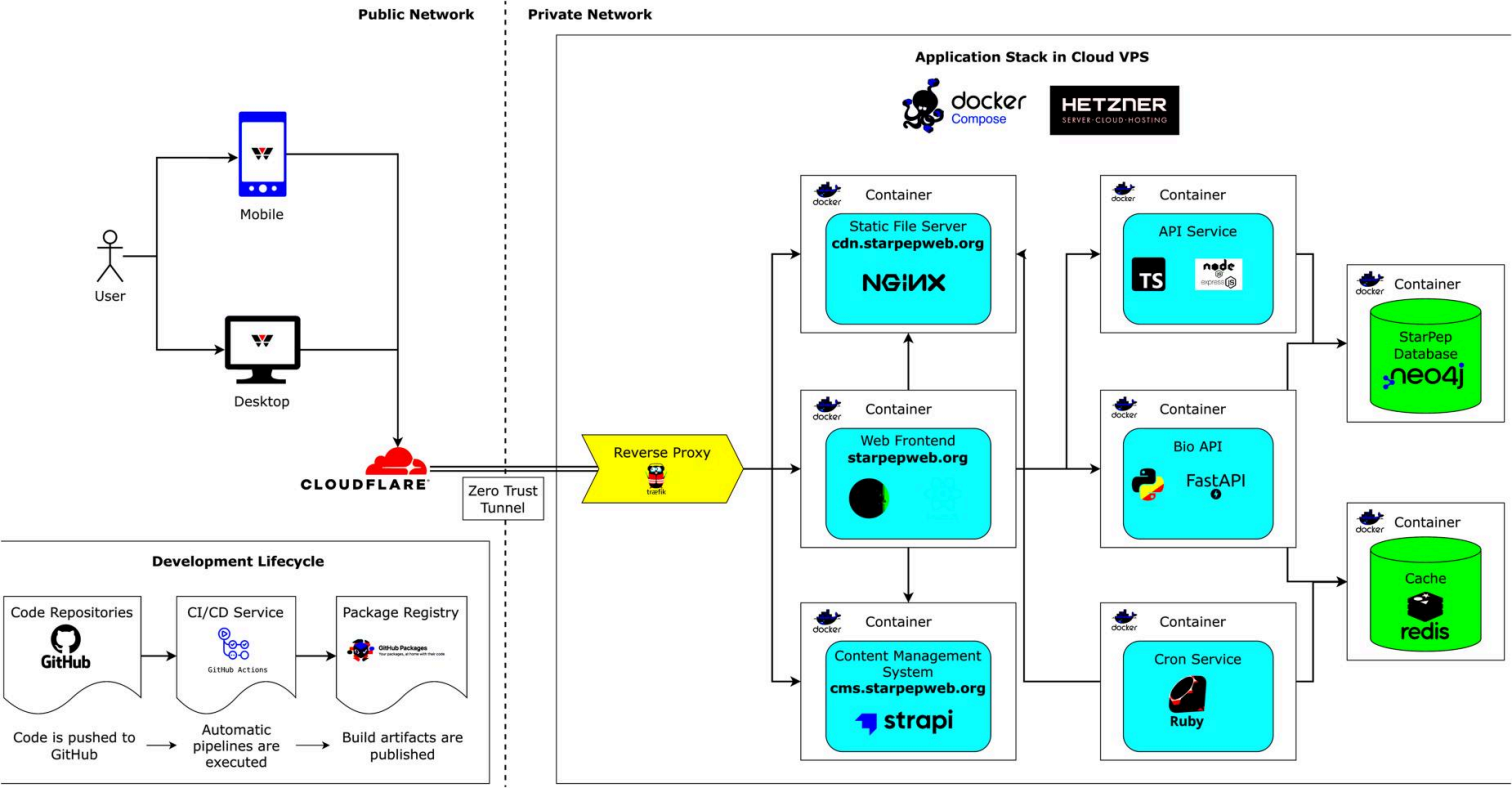
StarPepWeb system architecture. User requests arrive via a Cloudflare Zero Trust tunnel to a private Hetzner VPS where containers run under Docker Compose. A reverse proxy exposes only: (i) the StarPepWeb app (UI/API), (ii) a static file server for versioned downloads (datasets, nr subsets, embeddings, ESMFold models, Neo4j dump), and (iii) a content management system. Internal APIs and the Neo4j database remain private and unreachable from the internet. Arrows show browser→tunnel→proxy→public services and public↔internal flows, illustrating separation of public endpoints from internal components for security and scalability.

StarPepWeb is a modular, cloud-hosted platform of containerized microservices. Using Docker Compose on a virtual private server (VPS) enables reproducible deployments, easy scaling/updates and clean separation of public versus internal services (Fig. 1).

The frontend uses React/Next.js to deliver a responsive interface with efficient rendering and asset optimization, enabling dynamic filtering, exploration and downloads of peptide data (predicted 3D structures and numerical descriptors). Visual analytics use Chart.js for statistical plots, and 3Dmol.js for molecular visualization (Rego and Koes 2015).

Backend services are functionally separated. Python/FastAPI runs computational pipelines (e.g. sequence alignment) using Biopython (Cock *et al.* 2009). A Node.js/Express layer handles routing and API aggregation and interfaces with the Neo4j graph database that stores peptide entities and relationships. Redis caches repeated alignment/similarity results to reduce latency and load. Nginx serves static assets, Strapi manages administrative content, and scheduled cron tasks (Ruby scripts) periodically purge temporary user files. This split supports low-latency interactive queries while keeping compute isolated from public endpoints (Fig. 1).

Secure access and routing are handled by a reverse proxy (Traefik), which directs incoming traffic to the appropriate microservices by host/path while exposing only public endpoints and keeping internal APIs/Neo4j private (Fig. 1). Continuous integration and deployment with GitHub Actions automate builds, testing and rollouts, ensuring reproducible updates and rapid recovery.

## 3 Web interface

StarPepWeb is an integrated, browser-accessible platform for exploring, analyzing, and retrieving structurally annotated bioactive peptides. Its user-friendly interface comprises four main sections: “Search,” “Statistics,” “Downloads,” and “GitHub,” to support both experts and non-specialists.

### 3.1 Search module

Metadata-driven filtering by sequence length, origin (e.g. producer organism or synthetic), function (e.g. antimicrobial, antitumoral), target organisms or cell lines, sequence modifications, and physicochemical descriptors (e.g. hydropathicity, charge, isoelectric point, Boman index). Results appear in an exportable table, with options to download peptide sequences (FASTA), metadata, physicochemical properties, iFeature descriptors (AAC, DPC and GAAC), ESM-2 embeddings, and predicted 3D structures (PDB).

**Sequence-based similarity searches**:

Single-Query: Performs global (Needleman–Wunsch) (Needleman and Wunsch 1970) or local (Smith–Waterman) (Smith and Waterman 1981) alignments against StarPepDB, with user-selectable substitution matrices (e.g. BLOSUM, PAM) and similarity thresholds.
Multi-Query: Simultaneous comparison of multiple queries/peptides against the full database using pairwise alignment scores between each query and candidate, followed by the calculation of a fusion score (using maximum, minimum, or average aggregation) per candidate. Hits above the user-defined threshold are returned and ranked accordingly, improving sensitivity to peptide families that share functional motifs (Castillo-Mendieta *et al.* 2024).

### 3.2 Statistics

General Information: Summaries of peptide counts and non-standard amino acid occurrences; distributions by functional class and source databases; and database-similarity heatmap; and an interactive AAC plot by database, function and origin.
Metadata: Frequency plots by length, functional class, source, target origin and sequence terminal modifications.
Features: Interactive bar plots of physicochemical property distributions visualizations (Chart.js). Pairwise scatter plots are also visualized to facilitate multivariate pattern analysis.

### 3.3 Downloads section

Versioned, curated datasets comprising the complete StarPepDB snapshot (45 120 non-redundant peptides); ESMFold-predicted 3D structures (PDB); physicochemical features and molecular descriptors; ESM-2 embeddings; and iFeature-derived descriptors (AAC, DPC, GAAC) generated offline—all provided as precomputed files. Also available are non-redundant subsets at sequence-identity thresholds 0.90–0.50, datasets for each of the 40 integrated databases (full content and nr 0.90/0.50 subsets), and the complete Neo4j graph database for offline clustering and network-mining analyses.

### 3.4 GitHub section

The project’s source code, technical documentation, version updates, and installation guidelines are available (see Availability), promoting transparency, reproducibility, and community contributions.

## 4 Discussion and conclusions

StarPepWeb contributes to the in silico study of bioactive peptide by integrating a comprehensive, curated and nr database enriched with per-entry metadata, ESMFold-predicted 3D structures and numerical descriptors from ESM-2 and iFeature (Chen *et al.* 2018, Lin *et al.* 2023). This integration enables sequence- and structure-based analyses, that go beyond resources focused solely on sequences or metadata.

The platform addresses key challenges in peptide informatics by offering a unified web interface for filtering, comparing, and analyzing large-scale peptide datasets. In particular, the multi-query similarity search can reveal peptides sharing functional or structural features that may be missed by conventional single-sequence alignment approaches.

Built on a modular, container-based architecture, StarPepWeb ensures scalability and ease of maintenance, facilitating periodic database updates and the integration of new computational modules. The design also supports extension to additional peptide or protein databases, providing a flexible scaffold for applications.

StarPepWeb promotes transparency and reproducibility by providing public access to curated and computed datasets, as well as its GitHub code repository. By design, the platform is data-centric: it prioritizes uniform, platform-wide representations and reproducible, versioned exports over in-platform predictors. As a result, version 1 omits built-in ADMET/machine learning (ML) classifiers, interactive clustering and server-side embedding search; these analyses are run offline using the provided datasets.

Future enhancements will prioritize optional ML-based classifiers, support for user-uploaded sequences, and an ANN-based embedding-search endpoint (server-side vector index) to complement alignment-based searches. Advanced modules for ligand–receptor interaction and ADMET will also be exposed via a stable API. Overall, StarPepWeb provides a robust and extensible environment for the integrative analysis of peptide sequences, structures, and properties, serving as a valuable resource for researchers in computational biology and peptide therapeutics.

## Data Availability

The data underlying this article are available in StarPepWeb at https://starpepweb.org, with versioned curated datasets accessible through the *Downloads* section. Source code, documentation, and precomputed descriptors (ESM-2 embeddings, iFeature features, and ESMFold models) are available in the GitHub repository at https://github.com/starpep-web.

## References

[vbaf261-B1] Agüero-Chapin G , Antunes A, Mora JR et al Complex networks analyses of antibiofilm peptides: an emerging tool for next-generation antimicrobials’ discovery. Antibiotics (Basel) 2023;12:747. 37107109 10.3390/antibiotics12040747 PMC10135022

[vbaf261-B2] Aguilera-Mendoza L , Marrero-Ponce Y, Beltran JA et al Graph-based data integration from bioactive peptide databases of pharmaceutical interest: toward an organized collection enabling visual network analysis. Bioinformatics 2019;35:4739–47. 30994884 10.1093/bioinformatics/btz260

[vbaf261-B3] Akbarian M , Khani A, Eghbalpour S et al Bioactive peptides: synthesis, sources, applications, and proposed mechanisms of action. Int J Mol Sci 2022;23:1445. 35163367 10.3390/ijms23031445 PMC8836030

[vbaf261-B4] Al Musaimi O. FDA's stamp of approval: unveiling peptide breakthroughs in cardiovascular diseases, ACE, HIV, CNS, and beyond. J Pept Sci 2024;30:e3627. 38885943 10.1002/psc.3627

[vbaf261-B5] Ayala-Ruano S , Marrero-Ponce Y, Aguilera-Mendoza L et al Network science and group fusion similarity-based searching to explore the chemical space of antiparasitic peptides. ACS Omega 2022;7:46012–36. 36570318 10.1021/acsomega.2c03398 PMC9773354

[vbaf261-B6] Cabas-Mora G , Daza A, Soto-García N et al Peptipedia v2.0: a peptide sequence database and user-friendly web platform. A major update. Database (Oxford) 2024;2024:baae113. 10.1093/database/baae113 PMC11734279 39514414

[vbaf261-B7] Castillo-Mendieta K , Agüero-Chapin G, Marquez E et al Multi-query similarity searching models: an alternative approach for predicting hemolytic activity from peptide sequence. Chem Res Toxicol 2024;37:580–9. 38501392 10.1021/acs.chemrestox.3c00408

[vbaf261-B8] Chen Z , Zhao P, Li F et al iFeature: a Python package and web server for features extraction and selection from protein and peptide sequences. Bioinformatics 2018;34:2499–502. 29528364 10.1093/bioinformatics/bty140 PMC6658705

[vbaf261-B9] Cock PJA , Antao T, Chang JT et al Biopython: freely available Python tools for computational molecular biology and bioinformatics. Bioinformatics 2009;25:1422–3. 19304878 10.1093/bioinformatics/btp163 PMC2682512

[vbaf261-B10] Das D , Jaiswal M, Khan FN et al PlantPepDB: a manually curated plant peptide database. Sci Rep 2020;10:2194. 32042035 10.1038/s41598-020-59165-2 PMC7010657

[vbaf261-B11] de Jong A , van Hijum SAFT, Bijlsma JJE et al BAGEL: a web-based bacteriocin genome mining tool. Nucleic Acids Res 2006;34:W273–9. 16845009 10.1093/nar/gkl237 PMC1538908

[vbaf261-B12] de Llano García D , Marrero-Ponce Y, Agüero-Chapin G et al Innovative alignment-based method for antiviral peptide prediction. Antibiotics (Basel) 2024;13:768. 39200068 10.3390/antibiotics13080768 PMC11350826

[vbaf261-B13] Di Luca M , Maccari G, Maisetta G et al BaAMPs: the database of biofilm-active antimicrobial peptides. Biofouling 2015;31:193–9. 25760404 10.1080/08927014.2015.1021340

[vbaf261-B14] Hammami R , Ben Hamida J, Vergoten G et al PhytAMP: a database dedicated to antimicrobial plant peptides. Nucleic Acids Res 2009;37:D963–8. 18836196 10.1093/nar/gkn655 PMC2686510

[vbaf261-B15] Hammami R , Zouhir A, Le Lay C et al BACTIBASE second release: a database and tool platform for bacteriocin characterization. BMC Microbiol 2010;10:22. 20105292 10.1186/1471-2180-10-22 PMC2824694

[vbaf261-B16] Jhong J-H , Yao L, Pang Y et al dbAMP 2.0: updated resource for antimicrobial peptides with an enhanced scanning method for genomic and proteomic data. Nucleic Acids Res 2022;50:D460–70. 34850155 10.1093/nar/gkab1080 PMC8690246

[vbaf261-B17] Kapoor P , Singh H, Gautam A et al TumorHoPe: a database of tumor homing peptides. PLoS One 2012;7:e35187. 22523575 10.1371/journal.pone.0035187 PMC3327652

[vbaf261-B18] Lin Z , Akin H, Rao R et al Evolutionary-scale prediction of atomic-level protein structure with a language model. Science 2023;379:1123–30. 36927031 10.1126/science.ade2574

[vbaf261-B19] Liu L , Yang L, Cao S, et al CyclicPepedia: A knowledge base of natural and synthetic cyclic peptides. Brief Bioinform 2024;25:bbae190. 38678388 10.1093/bib/bbae190 PMC11056021

[vbaf261-B20] Mulvenna JP , Wang C, Craik DJ. CyBase: a database of cyclic protein sequence and structure. Nucleic Acids Res 2006;34:D192–4. 16381843 10.1093/nar/gkj005 PMC1347368

[vbaf261-B21] Needleman SB , Wunsch CD. A general method applicable to the search for similarities in the amino acid sequence of two proteins. J Mol Biol 1970;48:443–53. 5420325 10.1016/0022-2836(70)90057-4

[vbaf261-B22] Novkovic M et al DADP: the database of anuran defense peptides. Bioinformatics 2012;28:1406–7. 22467909 10.1093/bioinformatics/bts141

[vbaf261-B23] Pirtskhalava M , Amstrong AA, Grigolava M et al DBAASP v3: database of antimicrobial/cytotoxic activity and structure of peptides. Nucleic Acids Res 2021;49:D288–97. 33151284 10.1093/nar/gkaa991 PMC7778994

[vbaf261-B24] Qin D , Bo W, Zheng X et al DFBP: a comprehensive database of food-derived bioactive peptides for peptidomics research. Bioinformatics 2022;38:3275–80. 35552640 10.1093/bioinformatics/btac323

[vbaf261-B25] Rego N , Koes D. 3Dmol.js: molecular visualization with WebGL. Bioinformatics 2015;31:1322–4. 25505090 10.1093/bioinformatics/btu829 PMC4393526

[vbaf261-B26] Romero M , Marrero-Ponce Y, Rodríguez H et al A novel network science and similarity-searching-based approach for discovering potential tumor-homing peptides from antimicrobials. Antibiotics (Basel) 2022;11:401. 35326864 10.3390/antibiotics11030401 PMC8944733

[vbaf261-B27] Shi G , Kang X, Dong F et al DRAMP 3.0: an enhanced comprehensive data repository of antimicrobial peptides. Nucleic Acids Res 2022;50:D488–96. 34390348 10.1093/nar/gkab651 PMC8728287

[vbaf261-B28] Smith TF , Waterman MS. Identification of common molecular subsequences. J Mol Biol 1981;147:195–7. 7265238 10.1016/0022-2836(81)90087-5

[vbaf261-B29] Tyagi A , Tuknait A, Anand P et al CancerPPD: a database of anticancer peptides and proteins. Nucleic Acids Res 2015;43:D837–43. 25270878 10.1093/nar/gku892 PMC4384006

[vbaf261-B30] Waghu FH , Barai RS, Gurung P et al CAMPR3: a database on sequences, structures and signatures of antimicrobial peptides. Nucleic Acids Res 2016;44:D1094–7. 26467475 10.1093/nar/gkv1051 PMC4702787

[vbaf261-B31] Wang G , Li X, Wang Z. APD3: the antimicrobial peptide database as a tool for research and education. Nucleic Acids Res 2016;44:D1087–93. 26602694 10.1093/nar/gkv1278 PMC4702905

